# Interleukin 13 receptor alpha 2 directed immunotherapy targets hepatic fibrosis and tumorigenesis induced by choline deficient diet in an in vivo mouse model of nonalcoholic steatohepatitis

**DOI:** 10.1186/2051-1426-2-S3-P182

**Published:** 2014-11-06

**Authors:** Toshio Fujisawa, Bharat H Joshi, Raj K Puri

**Affiliations:** 1 NTT Medical Center Tokyo, Department of Gastroenterology, Tokyo, Japan; 2 Division of Cellular and Gene Therapies, Center for Biologics Evaluation and Research, FDA, Bethesda, MD, USA

## 

Nonalcoholic fatty liver disease is one of the most common causes of chronic liver disease in the United States. It is estimated that 3% of US population has nonalcoholic steatohepatitis (NASH). NASH can progress to cirrhosis leading to development of hepatocellular carcinoma. Previously, we have demonstrated that hepatic stellate cells (HSC) in sinusoidal lesions of liver in patients with NASH express high levels of high-affinity Interleukin-13R alpha2 (IL-13Ra2), which is co-localized with smooth muscle actin. In contrast, fatty liver and normal liver specimens do not express IL-13Ra2. To simulate human hepatic fibrotic disease, we also developed NASH in rats by feeding a choline-deficient L-amino acid diet. These rats developed liver fibrosis and increased liver enzymes in the periphery [1]. Here we report generation of another model of NASH by feeding a choline-deficient (CDAA) diet to mice. CDAA fed mice developed significant liver fibrosis as evident by hydroxyproline assay and collagen-1 mRNA expression. Interestingly, these mice also developed macroscopic liver tumors in a long term follow-up study. Existence of tumors was confirmed histologically. CDAA mouse livers overexpressed IL-13Ra2 chain at mRNA and protein levels but decreased serum albumin, increased alkaline phosphatase and alanine aminotransferase but total bilirubin values were unaffected. When these mice were treated with low doses of IL-13PE immunotoxin, we did not observe any decrease in fibrosis. However, they showed improved serum albumin and alkaline phosphatase levels, but had no effect on alanine aminotransferase and total bilirubin. When CDAA mice were treated with higher dose of IL13-PE (100 µg/kg/day), a significant decrease in fibrosis was observed. CDAA diet significantly increased TGF-β1 levels in the liver, which was also reversed by high dose IL-13PE treatment. Further IHC analyses in mouse liver specimens revealed that CDAA diet activated the hepatic stellate cells and increased TGF-β1 and IL-13Rα2 expression. IL-13PE decreased both TGF-β1 and IL-13Rα2 expression, but did not affect activation of the hepatic stellate cells. Additional studies are ongoing to examine the effect of IL-13-PE on liver tumor development. These results indicate that IL-13Rα2 targeted immunotherapeutic approaches either alone or in combination with other therapeutic agents may provide viable options for therapy of NASH and prevention of development of hepatocellular carcinoma.

## References

[B1] Shimamura T Fujisawa T Husain SR Kioi M Nakajima A Puri RK Novel role of IL-13 in fibrosis induced by nonalcoholic steatohepatitis and its amelioration by IL-13R-directed cytotoxin in a rat model J Immunol 2008 181 7 4656 65 10.4049/jimmunol.181.7.4656 18802068

